# Objectively Measured Daily Steps and Subsequent Long Term All-Cause Mortality: The Tasped Prospective Cohort Study

**DOI:** 10.1371/journal.pone.0141274

**Published:** 2015-11-04

**Authors:** Terence Dwyer, Angela Pezic, Cong Sun, Jenny Cochrane, Alison Venn, Velandai Srikanth, Graeme Jones, Robin Shook, Xuemei Sui, Andrew Ortaglia, Steven Blair, Anne-Louise Ponsonby

**Affiliations:** 1 The George Institute for Global Health, Oxford, United Kingdom; 2 Menzies Research Institute, University of Tasmania, Hobart, Tasmania, Australia; 3 Murdoch Childrens Research Institute, Royal Children’s Hospital, Melbourne, Victoria, Australia; 4 Department of Paediatrics, University of Melbourne, Melbourne, Victoria, Australia; 5 Stroke and Ageing Research Group, Medicine, School of Clinical Sciences, Monash University, Melbourne, Victoria, Australia; 6 Iowa State University of Science and Technology, Ames, Iowa, United States of America; 7 Department of Exercise Science, Arnold School of Public Health, University of South Carolina, Columbia, South Carolina, United States of America; 8 Department of Epidemiology and Biostatistics, Arnold School of Public Health, University of South Carolina, Columbia, South Carolina, United States of America; Nathan Kline Institute and New York University School of Medicine, UNITED STATES

## Abstract

**Background:**

Self–reported physical activity has been inversely associated with mortality but the effect of objectively measured step activity on mortality has never been evaluated. The objective is to determine the prospective association of daily step activity on mortality among free-living adults.

**Methods and Findings:**

Cohort study of free-living adults residing in Tasmania, Australia between 2000 and 2005 who participated in one of three cohort studies (n = 2 576 total participants). Daily step activity by pedometer at baseline at a mean of 58.8 years of age, and for a subset, repeated monitoring was available 3.7 (SD 1.3) years later (n = 1 679). All-cause mortality (n = 219 deaths) was ascertained by record-linkage to the Australian National Death Index; 90% of participants were followed-up over ten years, until June 2011. Higher daily step count at baseline was linearly associated with lower all-cause mortality (adjusted hazard ratio AHR, 0.94; 95% CI, 0.90 to 0.98 per 1 000 steps; *P* = 0.004). Risk was altered little by removing deaths occurring in the first two years. Increasing baseline daily steps from sedentary to 10 000 steps a day was associated with a 46% (95% CI, 18% to 65%; *P* = 0.004) lower risk of mortality in the decade of follow-up. In addition, those who increased their daily steps over the monitoring period had a substantial reduction in mortality risk, after adjusting for baseline daily step count (AHR, 0.39; 95% CI, 0.22 to 0.72; *P* = 0.002), or other factors (AHR, 0.38; 95% CI, 0.21–0.70; *P* = 0.002).

**Conclusions:**

Higher daily step count was linearly associated with subsequent long term mortality among free living adults. These data are the first to quantify mortality reductions using an objective measure of physical activity in a free living population. They strongly underscore the importance of physical inactivity as a major public health problem.

## Introduction

The inverse associations between physical activity (PA) and cardiovascular disease (CVD), [1–3] some cancers, [4] and all-cause mortality [5–7] have been demonstrated using self-reported PA. A meta-analysis concluded that the findings are consistent across studies and that there is a dose-response relationship between self-reported PA and mortality. [8] Parallel studies of change in fitness and mortality outcomes also indicate a likely benefit of PA on mortality. [9–11] Randomised trials involving PA interventions have demonstrated beneficial effects on elevated HDL cholesterol [12] and increased insulin sensitivity [13] but have not been maintained long enough to evaluate mortality. Prospective studies with accurate objective measurements of physical activity in a free-living population that can then quantify subsequent mortality risk have not been available.

Developments in technology to improve the objective PA measurement in humans through the use of objective measures such as pedometers and accelerometers have promise. [14,15] Pedometers capture most of the variance in PA measured by accelerometers in adult populations. [16,17] Data from cohort studies using pedometers are now starting to emerge. [18] Yates et al, using data from the NAVIGATOR trial, [18] estimated a 10% reduction in annual cardiovascular disease (CVD) event rate for a 2 000 daily step increase at baseline. However, the NAVIGATOR study participants had impaired glucose tolerance and CVD or higher CVD risk so extrapolation to healthy free-living populations is difficult. Further, the level of PA was low compared to some general population samples: 5 000 daily steps [18] compared to up to 9 000 steps found in general populations aged over 50 years. [19]

The recent U.S. nationally representative National Health and Nutrition Examination Survey (NHANES) used accelerometers to focus on sedentary behaviour and reported that those who were in the highest category of sedentary time had an odds ratio (OR) for all-cause mortality of 5.0 over a 2.8 year follow-up period compared to the least sedentary. [20] This suggests that an even greater effect might be found in healthier populations.

The possibility of reverse causality also needs to be addressed. The removal of early deaths in analyses of cohort data has been recognised as a critical way to take into account the possibility of reverse causality. [21] Neither of the above studies had sufficient follow-up time to address this. [18,20]

In this report, we pool three population-based adult cohorts based in Tasmania [21–24] to enable us to examine the prospective association between daily step activity and all-cause mortality over a decade of follow-up. The independent association between a temporal objective change in daily steps and mortality is also assessed.

## Methods

### The Australian Diabetes, Obesity and Lifestyle (AUSDIAB) study

#### Participants

The AUSDIAB study was a population based study designed to investigate diabetes. [25] Baseline testing was conducted on participants over 18 years of age in 2000. There were 1 136 participants who had a baseline oral glucose tolerance test then wore pedometers for two days at baseline and 642 provided pedometer data at both baseline and at a repeat interview. [21]

#### Measures

Information on PA was collected using the Active Australia Questionnaire. [26] A weighted sum of the responses (with vigorous-intensity activity given double-weighting) was calculated to quantify total hours of PA. [26] Pedometer data (Omron HJ-003 and Omron HJ-102; Omron Healthcare, Inc., Bannockburn, IL) [21] were collected.

#### Ethics

All participants gave informed written consent and the AUSDIAB was approved by the Southern Tasmanian Health and Medical Human Research Ethics Committee, University of Tasmania.

### The Tasmanian Older Adult Cohort (TASOAC) study

#### Participants

The TASOAC study was a prospective study. Individuals between the ages of 50 and 80 years were randomly selected from the electoral roll in Southern Tasmania. Of all initially eligible individuals, 1 041 attended a baseline clinic between March 2002 and September 2004. Repeat interview data were collected for 793 eligible participants at a subsequent clinic approximately two to three years later.

#### Measures

PA was assessed objectively as steps per day, as determined by pedometer at baseline (Omron HJ-003 and HJ-102; Omron Health-care Kyoto, Japan) and at second interview (Yamax Digi-Walker SW-200) as previously described. [27] PA was also recorded by questionnaire using the International Physical Activity Questionnaire (IPAQ). [28]

#### Ethics

All participants gave informed written consent and the TASOAC was approved by the Southern Tasmanian Health and Medical Human Research Ethics Committee, University of Tasmania.

### The Tasmanian Study of Cognition and Gait (TASCOG) study

#### Participants

Non institutionalised residents from southern Tasmania aged between 60 and 86 years (n = 399) were randomly selected from the electoral roll. The collection of baseline measures commenced in 2005.

#### Measures

For PA, the average number of steps per day was measured using a Yamax Digi-Walker SW-200 pedometer at baseline and at second review worn for seven days. [29] PA was also collected by questionnaire using the IPAQ. [28]

#### Ethics

All participants gave informed written consent and the TASCOG was approved by the Southern Tasmanian Health and Medical Human Research Ethics Committee, University of Tasmania.

### The Tasped cohort

The Tasped cohort was developed by pooling three pre-existing cohort studies based on Tasmanian adults–the Australian Diabetes Obesity and Lifestyle (AUSDIAB) study, [21,22] the Tasmanian Older Adult Cohort (TASOAC) study [23] and the Tasmanian Study of Cognition and Gait (TASCOG) study. [24] The Tasped cohort participants were linked to the National Death Index, compiled by the Australian Institute of Health and Welfare for all Australian deaths, to determine mortality up to June 2011. [30] In all studies, the self-report of lifestyle factors, medications and medical history including doctor diagnosed conditions was recorded by questionnaire. In all studies, a self-administered validated food frequency questionnaire (FFQ), developed by the Cancer Council of Victoria, was used to assess dietary and alcohol intake during the previous 12 months. [31] The Southern Tasmanian Health and Medical Human Research Ethics Committee University of Tasmania, specifically approved the Tasped cohort.

### Statistical Analysis

The primary analysis was done with data from all participants who completed baseline PA measures: questionnaire and pedometer-based. The summary PA measure for each period was the average daily number of steps over two consecutive days where at least one was a weekday. We have previously established this measure of daily steps is inversely and prospectively associated with hypertension (AOR 0.87 (95% CI 0.77–0.97)) [22] and insulin sensitivity and adiposity in free-living adults. [21]

Time-to-event analyses were used to assess the relationship between PA and all-cause mortality. The Kaplan-Meier method was used to estimate unadjusted survival curves by PA group. The Cox proportional hazards model [32] was used to estimate crude and adjusted hazard ratios (HRs) controlling for potential confounders as listed in Table footnotes and text. The proportionality assumption was assessed graphically via log-log survival curves and deemed to be appropriate. HRs and 95% confidence intervals (CIs) accounted for age and sex unless those factors were the parameters of interest. Tests of trend of categorical variables were undertaken by using the categorical variable as a linear predictor, providing a *P*-value for the Wald test associated with the average HR per category increase. The linearity of the continuous exposures, such as baseline daily steps, was assessed using fractional polynomials. [33] In past work comparing the Omron to the Yamax pedometer, the two types were found to be linearly correlated (r = 0.88; *P* < 0.001, with the Omron reading higher by a mean difference of 9%). [29] We therefore multiplied the Omron pedometers for the correction factor (0.91) found in the comparison study. [29] All analyses were performed using Stata version 13 (Stata Corp, College Station, Texas, USA).

## Results

### Characteristics of the pooled cohort

Among the 2 576 Tasped cohort participants, with an average age of 58.8 (SD 13.2) years at baseline, there were 219 deaths during a follow-up period where more than 90% were followed for more than 10 years, to June 2011. The average annual mortality rate during cohort follow-up was 8.5 (95% CI, 7.4–9.6) per 100 person-years. Table 1 shows that participants had a high average daily step count. Mortality risk was higher for men than women (HR, 1.65; 95% CI, 1.26–2.17; *P* <0.0001) and increased by 1.08 (95% CI, 1.06–1.09; *P*< 0.0001) per year of age at cohort entry.

**Table 1:** Characteristics of the Pooled Cohort at Baseline and Repeat Interview, Mean (SD).

	Baseline Review		Repeat Review	
Characteristic	Males (N = 1 226)	Females (N = 1 350)	Males (N = 813)	Females (N = 866)
Age, years	59.8 (13.0)	57.8 (13.4)	63.6 (11.0)	62.0 (11.2)
Daily steps	8 781 (4.538)	8 925 (4.485)	7 880 (4 106)	7 774 (4 051)
Moderate physical activity in past weeks, hrs	3.0 (4.4)	2.5 (4.0)	4.3 (4.8)	3.4 (4.4)
Vigorous physical activity in past week, hrs	1.4 (2.9)	1.1 (2.7)	2.1 (3.5)	1.4 (2.9)
Waist circumference, cm	97.4 (11.5)	88.1 (13.7)	98.3 (11.1)	89.2 (13.1)
Waist to hip ratio	0.95 (0.07)	0.85 (0.08)	0.95 (0.07)	0.85 (0.08)
Weight, kg	82.8 (14.0)	72.2 (14.9)	83.2 (13.9)	72.7 (14.5)
Height, cm	173.5 (7.8)	162.4 (6.9)	172.8 (7.5)	161.6 (6.9)
Body mass index, kg/m^2^	27.5 (4.1)	27.4 (5.3)	27.7 (3.9)	27.7 (5.2)

### The prospective association between daily step activity at baseline and subsequent mortality

Table 2 displays the pattern of mortality for the quintiles of daily step activity, rounded to the nearest 500 steps. There was a significantly lower mortality rate for those in the higher quintiles of daily step activity at baseline (*P* trend < 0.001). Those in the lowest step activity had the highest mortality but it should be noted that these estimates are unadjusted and this group were also older. When this group was excluded, the trend for decreased mortality with increasing daily steps at cohort entry remained evident (Table 2).

**Table 2:** Distribution of Observed vs. Expected deaths from Cohort Entry (Baseline) Interview until 2011 Linkage, by Number of daily steps at Baseline.

Quintile	Number of steps	Observed	Expected	Ratio of O:E
1	0–5 550	101	50	2.02
2	5 551–8 000	49	54	0.91
3	8 001–10 000	29	38	0.76
4	10 001 to 13 500	28	45	0.62
5	13 501 to 39 164	12	32	0.38

Log-rank test for Equality of survivor functions, P < 0.001

Log-rank test for Equality of survivor functions excluding 0–5 550 category, P = 0.03

Table 3 shows the prospective association between baseline daily step count and mortality. Higher daily steps at baseline were associated with a lower risk of all-cause mortality with an AHR of 0.94 (95% CI, 0.90–0.98; *P* = 0.002) per 1 000 step increase. This prospective inverse association was evident within each cohort (AUSDIAB: AHR 0.95 (95% CI, 0.90–1.01) per 1 000 steps; TASOAC: AHR 0.91 (95% CI, 0.85–0.98) per 1 000 steps, TASCOG: AHR 0.85 (95% CI, 0.73–0.99) per 1 000 steps). Higher daily step activity at second review was also associated with lower mortality risk (Table 3). The 5 category model (Table 2) or fractional polynomial models did not fit the data better than a linear model, indicating a linear pattern was evident between increasing baseline daily steps and subsequent mortality in the adjusted analysis.

**Table 3:** The Association Between Daily Steps and All-cause Mortality.

	HR^a^ (95% CI)	*P* value	AHR^b^ (95% CI)	*P* value
Average daily steps at baseline, (per 1000 increase)	0.94 (0.90–0.98)	0.002	0.94 (0.90–0.98)	0.004
Average daily steps at baseline, (per 1 000 increase)^c^	0.92 (0.81–1.04)	0.20	0.92 (0.81–1.05)	0.22
Average daily steps at baseline, (per 1 000 increase)^d^	0.94 (0.90–0.98)	0.005	0.94 (0.90–0.99)	0.01
Average daily steps at repeat review, (per 1 000 increase)	0.90 (0.84–0.97)	0.004	0.90 (0.83–0.97)	0.007

^a^ Adjusted for age and sex

^b^ Adjusted for age, sex, BMI at baseline, total energy intake from all sources (kJ) at baseline, current smoking status at baseline, alcohol consumption (g/day) at baseline, education at baseline and study cohort

^c^ For deaths in the first two years

^d^ For later deaths excluding any deaths in the first two years after baseline

### Addressing possible reverse causality where recent premorbid illness may be responsible for reduced daily steps

We conducted a survival analysis where those who died in the first or second year after baseline measurements were excluded. The HR for daily step count and mortality for those who died two years or more after baseline was similar to that found in the full cohort (Table 3). To further assess the potential influence of co-morbidity and consequent reverse causality on the association between daily steps and mortality, we conducted a set of sub-group analyses which are shown in Table 4. The daily steps-mortality pattern did not differ substantially by age, sex, smoking status, obesity or a range of comorbidities (Table 4). The baseline average daily steps-mortality pattern in Table 3 persisted after individual adjustment for the comorbidities listed in Table 4.

**Table 4:** Difference in Effect of Baseline Daily Steps (Per 1,000 Step Increase) on Mortality by Participant Characteristics.

	Participants in group	Deaths		
Factor	No. (%)	No. (% of group)	HR (95% CI) ^a^	Heterogeneity *P* value^b^
Sex				
Male	1 226 (47.6)	136 (11.1)	0.92 (0.88–0.97)	
Female	1 350 (52.4)	83 (6.2)	0.96 (0.90–1.03)	0.30
Age				
Lower median age (25 to 60)	1 327 (51.5)	48 (3.6)	0.93 (0.87–0.99)	
Higher median age (61 to 86)	1 249 (48.5)	171 (13.7)	0.88 (0.84–0.93)	0.22
Smoking status ^a^				
Non-smoker	2 251 (87.6)	184 (8.2)	0.94 (0.90–0.98)	0.15
Current smoker	319 (12.4)	33 (10.3)	0.97 (0.89–1.07)	
Alcoholic drinks per day ^a^				
Lower than median (6.4) alcoholic drinks per day	1 257 (50.0)	123 (9.8)	0.92 (0.87–0.98)	
Higher alcoholic drinks per day (6.4 or more)	1 256 (50.0)	92 (7.3)	0.96 (0.91–1.02)	0.09
BMI status ^a^				
Normal weight	824 (32.1)	78 (9.5)	0.91 (0.85–0.98)	
Overweight	1 132 (44.1)	94 (8.3)	0.94 (0.88–1.00)	
Obese	612 (23.8)	45 (7.4)	0.91 (0.83–1.01)	0.93
Ever had stroke ^a^				
Yes	74 (4.8)	16 (21.6)	1.14 (0.78–1.68)	
No	1 456 (95.2)	124 (8.5)	0.97 (0.92–1.02)	0.15
Ever had myocardial infarction ^a^				
Yes	195 (7.6)	60 (30.8)	0.88 (0.80–0.98)	
No	2 370 (92.4)	157 (6.6)	0.98 (0.93–1.02)	0.11
Ever had hypertension ^a^				
Yes	930 (36.2)	108 (11.61)	0.95 (0.90–1.00)	
No	1 637 (63.8)	110 (6.7)	0.95 (0.90–1.00)	0.31
Ever had diabetes ^a^				
Yes	162 (6.3)	36 (22.2)	0.86 (0.75–1.00)	
No	2 410 (93.7)	183 (7.6)	0.96 (0.92–1.00)	0.13
Any risk factors ^a^ ^c^				
Yes	1 515 (69.9)	164 (10.8)	0.95 (0.90–0.99)	
No	652 (30.1)	35 (5.4)	0.89 (0.78–1.01)	0.25

^a^ HR takes age and sex into account

^**b**^ Difference in effect of baseline daily steps on mortality across categories of the factor.

^c^ Any risk factors, refers to any of the diseases listed in this Table.

### Comparison of the association between daily steps and self-reported physical activity time with subsequent mortality

First, we examined the correlation between step activity and self-reported activity. Daily step count was only weakly correlated with vigorous activity time per week (men; r = 0.11; *P* < 0.001, women: r = 0.12; *P* < 0.001). The correlations between daily step count and moderate activity time were even lower in magnitude (men: r = -0.03; *P* = 0.28; women: r = -0.06; *P* = 0.12). Table 5 shows that reported moderate and vigorous PA were also associated with reduced mortality. This was predominantly driven by the large category of 1326 people who reported doing no physical activity, with a test of trend across AHRs of 0.79 if this group were excluded. The association between daily steps and mortality was robust to this issue and did not differ markedly across categories of self-reported moderate or vigorous activity time, with *P* values for difference in effect of 0.60 and 0.76 respectively.

**Table 5:** Reported Physical Activity and Subsequent Mortality in the Tasped Cohort.

	Participants in group	Deaths (% in group)				
	No.	No. (%)	HR^a^ (95% CI)	*P* value	AHR^b^ (95% CI)	*P* value
0 hours	1 326	127 (9.6)	1 [Reference]		1 [Reference]	
0<2 hours	223	10 (4.5)	0.42 (0.22–0.81)	0.01	0.50 (0.25–0.96)	0.04
2<4 hours	301	20 (6.6)	0.53 (0.33–0.87)	0.01	0.60 (0.37–0.99)	0.05
4<7 hours	261	20 (7.7)	0.49 (0.30–0.80)	0.004	0.60 (0.35–1.00)	0.05
7 or more hours	433	37 (8.6)	0.45 (0.31–0.67)	<0.001	0.54 (0.36–0.82)	0.003
0 <3 hours	2 131	197 (9.2)	1 [Reference]		1 [Reference]	
3 or more hours	410	17 (4.2)	0.47 (0.28–0.77)	0.003	0.53 (0.31–0.90)	0.02

^a^ HR takes age and sex into account

^b^ Additionally adjusted for factors listed in Table 3

After mutual adjustment, both increasing moderate activity time (*P* < 0.001) and increasing daily steps (*P* = 0.02) were associated with lower mortality risk. Both daily step count (AHR, 0.94; 95% CI, 0.91–0.98 per 1 000 steps; *P* = 0.006) and 3 or more hours of vigorous exercise per week (AHR, 0.49; 95% CI, 0.30–0.82; *P* = 0.005) remained independently protective for subsequent mortality when they were included in the same model.

### Temporal change in daily steps over a monitoring period and mortality

**Fig 1:**
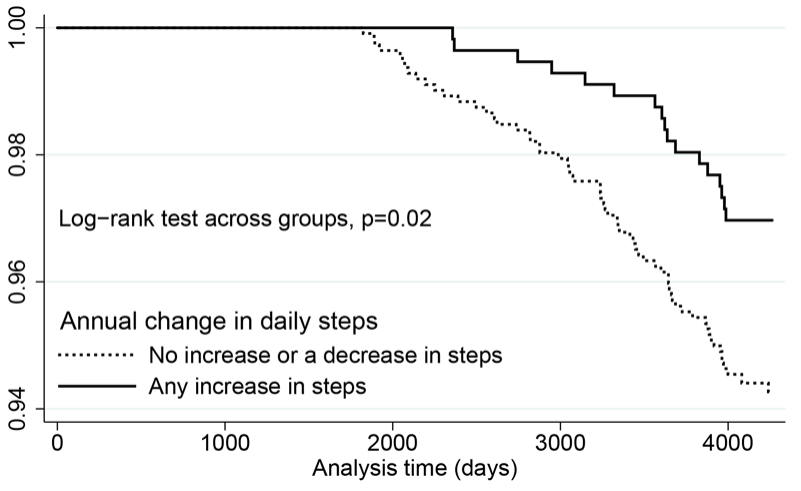
Kaplan-Meier probability estimates of survival by annual change in daily step activity from baseline to repeat interview, among Tasped participants with serial pedometer measures.

A subset of subjects participated in a repeat review (see Table 1), with a mean monitoring interval of 3.7 (SD 1.3) years after first review. Over this interval, a small average fall in steps was generally observed as the participants aged (annual median change in daily steps; -440 steps (interquartile range -1 367 to 285 steps). The correlation between daily steps at baseline and repeat interview was r = 0.55, p<0.001. Among those with repeat pedometer data and other measures, 5.6% (63/1 118) of those who had an annual average change in daily steps of none or less steps per day died, compared to with a mortality of 3.0% (17/561) for those who had an annual average increase in steps over time (*P* = 0.02) (Fig 1). Thus more deaths were observed than expected for those with a decline in steps over time (Table 6). The age and sex-adjusted hazard ratio for any annual daily increase or greater over the monitoring period was 0.49 (95% CI, 0.28–0.87; *P* = 0.01). Those with higher daily steps at first review were more likely to have a negative change (*P* < 0.001) over the monitoring period. Therefore, adjustment for baseline daily steps further increased the magnitude of the association between higher annual change in daily steps over the monitoring period and reduced mortality (AHR, 0.39; 95% CI, 0.22–0.72; *P* = 0.002). The association between any annual daily step increase over the monitoring period and mortality also persisted after further adjustment for change in BMI over the monitoring period (AHR, 0.32; 95% CI, 0.18–0.62, *P* = 0.001) or the baseline daily steps and the full set of confounders noted in Table 3 (AHR, 0.38; 95% CI, 0.21–0.70; *P* = 0.002).

**Table 6:** Distribution of Observed vs. Expected deaths from Repeat Interview until 2011 Linkage.

Group	Annual change in steps	Observed	Expected	Ratio of O:E
1	-4 700 to 0	63	53	1.19
2	1 to 2 820	17	27	0.63

Log-rank test for Equality of survivor functions, *P* = 0.02

### Mortality reduction associated with daily step increases

The relationship between baseline steps and subsequent mortality in the whole cohort was linear in nature. Thus, a sedentary person who takes a very low number of daily steps (<1 000) but who is able to change behaviour, for example, to meet the popular 10 000 daily step guideline [34] would have a 46% (95% CI, 18% to 65%) lower mortality risk but a sedentary person (<1 000 daily steps) who increased his or her steps for 3 000 steps for 30 minutes five days a week [35] would have a 12% (95% CI, 4% to 20%) reduction in mortality, based on the findings in Table 3. Thus, the application of physical activity guidelines such as not being sedentary but undertaking 10 000 steps a day [35] to the actual daily step–mortality patterns in this cohort study indicate a substantive magnitude of effect of PA on mortality.

## Discussion

The major finding from this pooled adult cohort study, comprising over 2 500 free-living participants followed-up over a decade, was that daily step count measured at baseline was inversely associated with all-cause mortality. Further, a temporal increase in annual daily steps was independently associated with lower mortality. These findings are the first concerning the association between objectively measured levels of PA and all-cause mortality in a free-living population. The association was independent of factors such as BMI or smoking. The observed effects were similar in magnitude of mortality benefit observed for statins in randomised trials. [36]

A major strength is that the objective daily steps–mortality findings permits a much clearer definition of the linear shape of the relationship. This explains why lower activity level was associated with an approximately 10% lower mortality but a greater magnitude change from sedentary to over 10 000 steps a day was associated with more than a 40% mortality reduction. These findings are consistent with the findings of a past meta-analysis on self-reported activity, where highly active men had a significantly lower mortality than moderately active men. [37] Here, we also validate a recent study also demonstrating a dose response effect of reported physical activity and mortality [38] by providing supportive objective findings. This concept of a dose-response relation between daily steps and mortality has important public health implications.

Although directly comparable studies are not yet available, this magnitude of effect is similar to the effect of daily step activity on cardiovascular events in the NAVIGATOR Study high CVD risk participants. [18] Also, although not directly comparable, this relatively large effect is consistent with the high likelihood of mortality for the highest category of sedentary time for all-cause mortality in the NHANES, with an odds ratio of five. [20] Otherwise, past large population based studies have generally used questionnaires that rely on the self-report of physical activity. One study that compared self-reported PA and exercise capacity simultaneously among the same cohort found a poor correlation between the two measures, though high levels of each were associated with reductions in mortality, [10] similar to the findings described here between self-reported PA and objectively measured PA. Reasons for the observed differences between objective and subjective methods to estimate PA typically focuses on biases that influence recall [39] or social desirability. [40] Also, a previous study reported that using pedometer significantly increased physical activity Therefore, a two-day pedometer may possibly overestimate daily physical activity compared to self-reported leisure-time physical activity [41].

The risk reduction was unlikely to be due to reverse causality. Removal of the first two years of deaths from the analysis did not alter the estimated HR. Intensity of PA was able to be considered and there was not a difference in magnitude of effect for daily step count with mortality among those taking vigorous activity versus those who reported not having done so. The inference that the inverse association between steps and death is not primarily due to concurrent illness influencing exercise tolerance was supported by the observation that the association between steps and mortality was not different among those with a reported doctor diagnosed illnesses. A potential limitation is that only two consecutive days of pedometer recording was used, but a previous study investigating how this might affect estimates reported that two days captured 89% of the variance over a seven day recording period. [42] It is reassuring that among the smaller sample in which repeat measures were also available, increasing steps over time was also found to be protective, even independently of baseline step count. Attrition bias is unlikely. Here, follow-up was for death, the National Death Index was used and has been shown to be relatively complete. [30]

Given that few longitudinal observations from healthy free-living populations with documented daily step activity and subsequent long-term disease or mortality exist, these findings are highly relevant for future pedometer-based PA promotion guidelines. Consideration should be given to the linear nature of the association found for daily steps and mortality. These findings strengthen the evidence base for the hypothesis that physical activity can play a substantial role in reducing mortality.

This report is the first to quantify the mortality reductions associated with objectively measured daily step activity in a free living population, and strongly underscore the importance of physical inactivity as a major public health problem.
