# Correction: Objectively Measured Daily Steps and Subsequent Long Term All-Cause Mortality: The Tasped Prospective Cohort Study

**DOI:** 10.1371/journal.pone.0146202

**Published:** 2015-12-31

**Authors:** Terence Dwyer, Angela Pezic, Cong Sun, Jenny Cochrane, Alison Venn, Velandai Srikanth, Graeme Jones, Robin P Shook, Xuemei Sui, Andrew Ortaglia, Steven Blair, Anne-Louise Ponsonby

The eighth author's name is spelled incorrectly. The correct name is: Robin P Shook.
